# Acute Right Ventricular Failure: Pathophysiology, Diagnostic Approach with Emphasis on the Role of Echocardiography

**DOI:** 10.2174/1573403X19666230206115611

**Published:** 2023-07-05

**Authors:** Han Naung Tun, Abdallah Almaghraby, Vladyslav Kavalerchyk, Denisa Muraru, Hatem Soliman-Aboumarie, Mahmoud Abdelnabi

**Affiliations:** 1 *Larner* College of Medicine’s UVM Medical Centre, University of Vermont, Burlington, Vermont, 05405, USA;; 2 Cardiology Department, Faculty of Medicine, Alexandria University, Alexandria, Egypt;; 3 Cardiology and Angiology Department, Helios Kliniken Schwerin, Schwerin, Germany;; 4 Department of Cardiovascular, Neural and Metabolic Sciences, Istituto Auxologico Italiano, IRCCS, S. Luca Hospital, Milan 20149, Italy;; 5 Department of Medicine and Surgery, University of Milano-Bicocca, Milan 20126, Italy;; 6 Department of Anaesthetics and Critical Care, Harefield Hospital, Royal Brompton and Harefield Clinical Group, Guy’s and St Thomas NHS Foundation Trust, Hill End Road, Uxbridge, London UB9 6JH, UK;; 7 Internal Medicine Department, Texas Tech University Health Sciences Center, Lubbock, Texas, USA

**Keywords:** Acute right, ventricular failure, echocardiography, management of acute right, prognosis, heart failure

## Abstract

Right ventricular function is one of the important predictors of survival in heart failure patients. In the past, there has been only limited knowledge regarding right-sided heart failure when compared to left-sided failure. However, there are more emerging data in recent years, and several studies have emphasized the unique features of the right ventricle regarding its anatomy, pathophysiology, clinical consequences, diagnostic modalities, and treatment options. Despite that, management of acute right ventricular failure is still challenging. This article summarizes an overview of acute right heart failure including pathophysiology, causes, clinical features, and diagnostic work-up with emphasis on the role of echocardiography.

## 1 INTRODUCTION

In simple terms, heart failure is a complex syndrome where the heart muscle cannot pump adequate blood to meet the body's requirements. In developed countries, the prevalence of heart failure ranges from 1-2% and it is growing as a result of prolonged life expectancy, increased surveillance, and longer survival of heart failure patients. The overall prevalence of heart failure in patients aged 65 years and over is about 12% [1]. However, it may be underestimated because the data registered only established cases. Heart failure can be classified into different types depending on left ventricular ejection fraction (heart failure with reduced ejection fraction (HFrEF) *vs.* heart failure with preserved ejection fraction (HFpEF)), duration (acute *vs.* chronic), ventricular involvement [left ventricular failure (LVF), right ventricular failure (RVF), biventricular heart failure (BVF)], and symptoms [New York Heart Association (NYHA) Class I, II, III, IV, or American College of Cardiology /American Heart Association (ACC/AHA) stage A, B, C, D] [2]. When a patient develops new-onset heart failure symptoms suddenly (de novo) or rapidly worsening of pre-existing chronic stable symptoms (within 24 hours duration), this is calledacute heart failure (AHF) [3]. Acute right heart failure (ARHF) accounts for 2.2 – 4.5% of patients admitted to hospitals with heart failure symptoms [4, 5]. Acute right ventricular failure (ARVF) is defined when there is radiological (electrocardiographic, echocardiographic) and biochemical (cardiac biomarkers) evidence of right ventricular dysfunction (RVD) plus clinical features of backward failure causing systemic congestion. In severe cases, inadequate blood flow from the right ventricle (RV) to pulmonary circulation can compromise left ventricular (LV) filling, thus reducing cardiac output and resulting in forward failure [6]. ARHF is immediately endangered to life requiring urgent medical intervention.

## 2 PATHOPHYSIOLOGY

In contrast to LV, RV has distinct anatomy and physiology. RV is triangular and crescent shaped. RV receives the systemic venous return and pumps blood through low-pressure, low-resistance pulmonary circulation. Although RV ejects the same stroke volume as LV, the stroke work of RV is about one-fourth of that of LV [7]. Thus, RV has a thinner wall with fewer muscle fibers. The systolic function of RV is mainly contributed (60%) by the contraction (shortening) of longitudinal muscle fibers. The traction of the RV free wall by LV contraction also contributes 20-40% of RV cardiac output [8]. The anatomical features of RV lead to its greater sensitivity to changes in afterload. A sudden rise in pulmonary artery pressure (PAP) (afterload), as in cases of massive pulmonary embolism (PE), is compensated by increased RV contractility *via* Frank-Starling law. However, the thin-walled RV musculature cannot adapt properly, and RV becomes acutely dilated, and subsequently, a significant decline in RV contraction occurs [3]. On the other hand, RV and LV share the same interventricular septum (ventricular interdependence). Acute RV dilatation shifts the septum to the left which impairs LV diastolic filling and contractility, and finally reduces cardiac output, hypotension, and ultimately, cardiogenic shock [9].

Causes of acute RV dysfunction can be categorized into 3 groups: acute increased afterload, acute volume overload, and reduced inotropy (Table **1**) [10]. Clinically, the most important etiologies of ARHF include:

1. Acute RV myocardial infarction accounts for one-third of inferior myocardial infarction (MI)

2. Acute massive pulmonary embolism (PE)

3. Acute respiratory distress syndrome (ARDS)

4. Iatrogenic [positive pressure ventilation, trauma caused by malposition of pacemaker electrodes, left ventricular assisted device (LVAD) insertion, associated with cardiothoracic surgery like postcardiotomy or after lung resection]

5. Sepsis

6. Acute valve dysfunction (tricuspid regurgitation)

7. Arrhythmia

## 3 CLINICAL FEATURES

Usually, patients with RHF in general, present with features of systemic congestion like elevated central venous pressure, hepatic congestion, ascites, and peripheral edema. A parasternal pansystolic murmur may be heard if there is associated tricuspid regurgitation. Right hypochondrial pain due to the congested liver has 80% specificity and 23% sensitivity for right-sided heart failure. Although not common, hepato-jugular reflux and ascites are specific clinical signs of right heart failure [11]. In severe forms of right heart failure, through interventricular dependence, LV diastolic dysfunction causes forward failure, thus, hypotension and low cardiac output. At that time, patients may present with systemic hypoperfusion symptoms such as lethargy and weakness [2].

## 4 DIAGNOSTIC WORK-UP

### 4.1 Laboratory Parameters

Brain natriuretic peptide (BNP) and N-terminal proBNP (NT-proBNP) are diagnostic and prognostic markers of heart failure. NT-proBNP has a longer half-life and is less affected by obesity than BNP, thus more used clinically. BNP level greater than 48.5 pmol/L indicates severe RV dysfunction (EF <30%) with high sensitivity (88%) and specificity (86%) [12]. Significant RV dysfunction is less likely with normal NT-proBNP levels. BNP levels of less than 100 pg/ml have a negative predictive value (NPV) of 90% [13]. Moreover, a higher BNP level (1415 pg/ml *vs*. 628 pg/ml) is associated with increased mortality in acute RVF [14]. Cardiac biomarkers such as troponin will be high in case of RVD due to PE or MI, having poor prognostic value [15]. Hyponatremia (Na ≤ 136mmol/L) is associated with advanced RHF and indicates poor survival in patients with pulmonary arterial hypertension as compared with normal sodium concentration [16]. Increased creatinine and C-reactive protein (CRP) levels also have prognosis significance in acute RHF [17].

### 4.2 Imaging Parameters

Chest radiography (CXR) and electrocardiography (ECG) can help in the diagnosis of ARHF although lack sensitivity. In ARHF, CXR may be completely normal with clear lung fields or may show cardiomegaly, pulmonary congestion, and associated cardiopulmonary diseases. There are no specific chest x-ray features for acute RVD. ECG may show the underlying cause of ARVF. ST-elevation (>1mm) in V3R and V4R indicates RV infarction which is present in 50% of inferior MI [9]. The classical ECG pattern of S1Q3T3 (large S wave in lead I, Q wave in lead III, inverted T in lead III) indicates acute pulmonary embolism (PE). S1Q3T3 changes were seen in 43% of acute non-massive PE and 70% of acute massive PE [18]. It has high specificity (90%) but low sensitivity (35%) [19]. Moreover, in cases of chronic RVF due to chronic pulmonary hypertension, there may be an RV strain pattern (RV hypertrophy and right axis deviation, RBBB). Cardiac magnetic resonance imaging (MRI) is the gold standard for identifying detailed cardiac anatomy and function. Cardiac CT can also be used to assess coronary arteries. However, ARHF patients usually present with tachycardia which makes cardiac computed tomography (CT) and MRI lower sensitivity. Although invasive, cardiac catheterization can measure right atrial pressure, cardiac index, pulmonary vascular resistance (PVR), and pulmonary artery impedance which can give valuable information regarding the diagnosis and prognosis of acute right heart failure [17].

### 4.3 Echocardiography

**Fig. (1):**
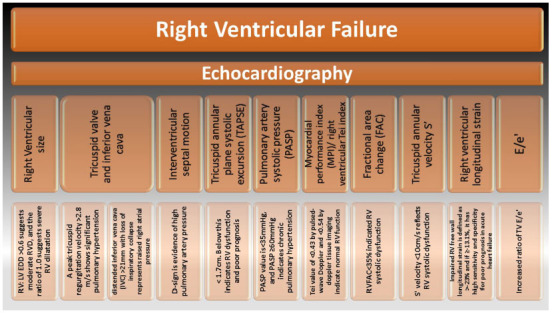
Echocardiographic parameters used in RV function assessment.

Due to the distinctive morphology of RV, echocardiographic assessment of detailed RV structure and function remains a challenge. The following parameters can be used to assess RV function (Fig. **1**):

• RV and RV size

• Tricuspid valve and inferior vena cava

• Interventricular septal motion

• Tricuspid annular plane systolic excursion (TAPSE)

• Pulmonary artery systolic pressure (PASP)

• Myocardial performance index (MPI)/ right ventricular Tei index

• Fractional area change (FAC)

• Tricuspid annular velocity S'

• RV longitudinal strain

• E/e′ ratio

• Three-dimensional volumes and right ventricular ejection fraction (RVEF)

### 4.4 Right Ventricular Size

An RV diameter >42 mm at the base and >35 mm at the mid-cavitary level indicates right ventricular dilatation [20]. Also, RV diameter is assessed in comparison with nearby structures. In the apical four-chamber (A4C) view on transthoracic echocardiography (TTE) or mid-esophageal four-chamber view on transesophageal echocardiography (TEE), RV: LV EDD >0.6 suggests moderate RVD, and the ratio of 1.0 suggests severe RV dilatation. In the parasternal long-axis (PLAX) view, RV size is approximately the same as that of the aorta and left atrium [21]. Dilated RV indicates high pulmonary artery pressure (PAP) and is associated with increased mortality.

### 4.5 Tricuspid Valve and Inferior Vena Cava

The RV tolerates volume overload better than pressure overload. A peak tricuspid regurgitation velocity >2.8 m/s shows significant pulmonary hypertension. In the sub-costal view, a distended inferior vena cava (IVC) >21mm with loss of inspiratory collapse represents increased right atrial pressure [9].

### 4.6 Abnormal Septal Motion

Dilated RV causes the interventricular septum (IVS) to shift towards the left ventricle during diastole (indicates volume overload) and systole (indicates pressure overload) [22]. This makes septal flattening and a D-shaped left ventricle. D-sign can be visualized on the parasternal short axis (PSAX) view at the mid-papillary level and is evidence of high pulmonary artery pressure [21].

### 4.7 Left Ventricle Size

LV size is one of the indirect measures of RV dysfunction. Small dynamic LV (LV internal end-diastolic diameter <4.0cm and LVEF > 60%) is associated with poor outcomes [23].

### 4.8 Tricuspid Annular Plane Systolic Excursion (TAPSE)

Tricuspid annular plane systolic excursion (TAPSE) is the simple and rapid indicator of RV longitudinal fibers' function and is well correlated with two-dimensional RVFAC and radionucleotide-derived RVEF. The normal reference limit of TAPSE is ≥1.7cm. Below this indicates RV dysfunction and poor prognosis [21].

### 4.9 Pulmonary Artery Systolic Pressure (PASP)

Pulmonary artery systolic pressure can be estimated on continuous wave (CW) Doppler echo by using the modified Bernoulli equation. PASP is a surrogate marker of RV afterload. The normal PASP value is <35mmHg, and PASP ≥60mmHg indicates chronic pulmonary hypertension.

### 4.10 Myocardial Performance Index (MPI)/ Right Ventricular Tei Index

MPI is sensitive in evaluating subclinical or early RV dysfunction. It is an echocardiographic Doppler index of combined systolic and diastolic function. It is calculated as RV isovolumic relaxation time plus isovolumic contraction time divided by pulmonary ejection time. Tei value of <0.43 by pulsed-wave Doppler and <0.54 by Doppler tissue imaging indicate normal RV function [20].

### 4.11 Fractional Area Change (FAC)

FAC is the ratio of change in the RV area (from diastole to systole) to the RV end-diastolic area. It is measured on apical four-chamber view on TTE or mid-esophageal four-chamber view on TEE. FAC is well correlated with MRI-derived ejection fraction. RVFAC <35% indicates RV systolic dysfunction [24].

### 4.12 Tricuspid Annular Velocity S'/ Systolic Excursion Velocity S'/ RV Tissue Doppler S' Velocity

It is easy to measure on an apical four-chamber view at the plane of the tricuspid annulus by using pulsed wave or color Doppler tissue imaging. S' velocity <10cm/s reflects RV systolic dysfunction [24].

### 4.13 E/e' Ratio

Like the left ventricle, RV diastolic function can be assessed by using RV- E/e', the ratio of trans-tricuspid valve early diastolic peak velocity (E) to early diastolic tricuspid annular tissue peak velocity (e'). RV diastolic dysfunction causes increased filling pressure; thus, the ratio of E/e' is increased [25]. Echocardiography is also valuable in the diagnosis of ARHF associated with acute massive PE. Echo evidence of RVD (like RV hypokinesia, RV pressure, and volume overload) may be detected if the pulmonary vascular obstruction is >30%. McConnell's sign (hypokinesia of RV free wall and base with normal RV apical motion due to acute RV pressure overload) has 94% specificity and 77% sensitivity in diagnosing pulmonary embolism [26].

### 4.14 Strain Echocardiography for Assessment of Right Ventricular Function

Speckle-tracking echocardiographic RV free wall longitudinal strain can detect subclinical RVD. It is calculated as the mean of the RV lateral, basal, mid, and apical segments. Impaired RV-free wall longitudinal strain is defined as >-23% and if ≥-13.1%, it has high sensitivity and specificity for poor prognosis in acute heart failure [27, 28]. In a study of heart failure patients with apparently normal RV systolic function (TAPSE >16 mm), abnormal RV-free wall strain was indicative of subclinical RVD and predictive of recurrent hospitalization and mortality [29]. RV-free wall strain has shown to be superior to conventional echocardiographic parameters in the detection of RV infarction in patients presenting with acute myocardial infarction [30]. Free wall longitudinal strain by 2D speckle tracking was predictive of functional capacity and 18-month mortality in a cohort of pulmonary arterial hypertension patients [31].

### 4.15 Stress Echocardiography in Right Ventricular Function

In a study that evaluated the feasibility of stress echocardiography in the assessment of RV contractile reserve comparing TAPSE, FAC, S-wave, systolic pulmonary artery pressure (sPAP), and right ventricle global longitudinal (free wall) strain (RVGLS) during baseline and at the peak stress showing that TAPSE, FAC, and S-wave are particularly feasible at rest, while TAPSE, S-wave, and sPAP are the most reliable measurements during RV stress echocardiography. The study concluded that RVGLS is a useful measurement in the assessment of RV contractile reserve [32]. The feasibility of velocity vector imaging (VVI) analysis to quantitatively assess RV function during stress echocardiography was studied where global RV function was estimated using RV ejection fraction while regional RV function was evaluated by radial velocity, circumferential strain, and strain rate (SR) of four segments from the mid-level RV short-axis view, and the longitudinal velocity, strain, and SR of six segments from the RV apical four-chamber view concluding that during stress echocardiography, global and regional RV function quantitative assessment was possible using VVI analysis with longitudinal velocity and SR of the RV basal lateral wall, which was significantly superior to longitudinal displacement or strain for detecting RV response during stress echocardiography [33]. Other studies have shown that stress echocardiography has a reasonable accuracy to diagnose exercise-induced pulmonary hypertension [34, 35]. Claessen *et al.* compared exercise echocardiography and exercise cardiac magnetic resonance imaging with simultaneous invasive pressure registration (ExCMRip) for the assessment of pulmonary vasculature and RV function. Echocardiographic parameters included mean PAP and sPAP, cardiac output (CO), FAC, TAPSE, and RV end-systolic pressure-area ratio as measures of RV contractile reserve concluding that RV echocardiographic parameters can be used as a feasible screening tool in the identification of pulmonary vascular disease in routine clinical practice [34]. van Riel *et al*. aimed to determine the noninvasive echocardiographic assessment of pulmonary pressures during exercise compared to simultaneous right heart catheterization (RHC) invasive measurements comparing tricuspid regurgitation (TR) Doppler estimation to invasive PAP measurement at risk and peak exercise concluding that in patients with high-quality TR Doppler signal, there was a positive correlation between echocardiographic parameters and invasive pulmonary pressure measures [35].

### 4.16 Three-dimensional Volumes and RVEF

Three-dimensional (3D) echocardiography allows a better anatomical definition of the RV compared to two-dimensional conventional echocardiography, including the base, the apex, and the outflow tract. Assessment of the RV with 3D-Echocardiography is feasible during routine standard echocardiography, the time needed for RV analysis (acquisition and off-line analysis) using 3D-Echocardiography is reasonably short, with a satisfactory quality of images [20].

3D-echocardiography of the RV is well-validated in many studies for the assessment of RV cardiomyopathy, atrial septal defect, Ebstein’s anomaly, and tetralogy of Fallot [36]. RVEF is an integrated result of the interaction between RV contractility and load, and therefore it does not directly reflect RV contractile function per se. So, 3D-echocardiography allows measurement of RVEF reflecting global RV systolic performance, with better sensitivity than 2D-echocardiography [20].

### 4.17 Acute Right Heart Failure *versus* Chronic Right Heart Failure

Some markers differentiate acute *versus* chronic RVF such as RV wall thickness, pulmonary artery systolic pressure (PASP), and 60/60 sign. RV end-diastolic wall thickness >5mm suggests right ventricular hypertrophy (RVH) which is rare in ARVF. PASP ≥40mmHg is indicative of pulmonary hypertension and PASP>60mmHg is generally consistent with chronic RVF. Again, the tricuspid insufficiency pressure gradient (∆P=4VTR Max2) and the pulmonary acceleration time (PAT) are used to calculate the 60/60 sign and if both values are less than 60, this is called the positive 60/60 sign and predicts ARHF. The positive 60/60 sign has 94% specificity in predicting ARVF associated with acute pulmonary embolism [37].

### 4.18 Right Ventricular Failure Management

**Fig. (2):**
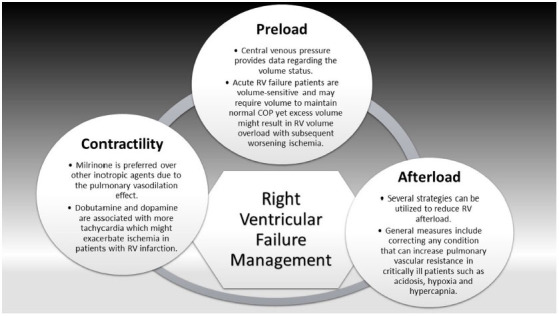
Right ventricular failure management.

The initial approach for the management of acute RV failure is based on the identification and correction of the underlying cause, reversal of the underlying etiology can be associated with better outcomes. A proper understanding of hemodynamics (preload, afterload, and contractility) is necessary for optimal management (Fig. **2**) [38].

### 4.19 Preload

Preload is essential to maintain cardiac output (COP). Central venous pressure (CVP) provides data regarding the volume status. Acute RV failure patients are volume-sensitive and may require volume to maintain normal COP yet excess volume might result in RV volume overload with subsequent worsening ischemia. Due to the negative effects of positive end-expiratory pressure (PEEP) on the preload, mechanical ventilation should be done cautiously in acute RV failure patients [38].

### 4.20 Afterload

Afterload optimization in patients with acute RV failure is necessary due to the failure of the RV to compensate for the acute changes in afterload. Several strategies can be utilized to reduce RV afterload. General measures include correcting any condition that can increase pulmonary vascular resistance (PVR) in critically ill patients such as acidosis, hypoxia (hypoxic induced pulmonary vasoconstriction), and hypercapnia. Lung protective mechanical ventilation strategies (lowest possible effective plateau pressure, tidal volume, and positive end-expiratory pressure) to prevent hypoxemia and hypercapnia and at the same time avoid any increase in pulmonary artery pressures which ultimately can help to optimize both RV preload and afterload [39]. Pulmonary vasodilators can also help to optimize RV afterload by relaxing pulmonary vascular smooth muscle with a resultant reduction of PA pressures and PVR. Inhaled nitric oxide (iNO) can be used to reduce the potential for V/Q mismatch. Short-term hemodynamic recovery has been associated with iNo use in patients with RV infarction [40].

### 4.21 Contractility

In acute RV failure, inotropes might be required for RV support to maintain preload. Milrinone is preferred over other inotropic agents due to the pulmonary vasodilation effect, but it should be used with caution due to the associated risk of hypotension. Dobutamine and dopamine are associated with more tachycardia which might exacerbate ischemia in patients with RV infarction. Mechanical support with an intraaortic balloon pump or a temporary right ventricular assist device (RVAD) might be used for RV support in RV infarction, cardiovascular surgery, or cardiac transplantation [38].

### 4.22 Prognosis

Data regarding the prognosis of acute RV failure is limited yet several reports stated poor outcomes of RV failure in patients with HFpEF, HFrEF, and pulmonary arterial hypertension (PAH) [41, 42].

## CONCLUSION

Acute right heart failure is not uncommon and is life-threatening if remains untreated without timely diagnosis and management. Despite advances in diagnostic modalities, detailed assessment of right ventricular structure and function remains challenging. Echocardiography is one of the most simple, reliable, and realistic tests for measuring RV function. RV systolic dysfunction can be assessed by TAPSE, MPI, FAC, and Tricuspid annular velocity S'. RV diastolic dysfunction can be estimated utilizing E/e'. RV wall thickness, PASP, and 60/60 signs are useful in differentiating acute right heart failure from chronic. Thus, clinicians should be knowledgeable in echocardiographic parameters of acute RV dysfunction which would guide the optimal management in acute care settings. The treatment of RV failure is mainly related to the underlying etiology, which should be reversed if possible.

## Figures and Tables

**Table 1:** Classification of acute RV failure.

**Acute increased afterload** • Acute massive pulmonary embolism • ARDS (due to hypoxic/ hypercapnic pulmonary vasoconstriction, pulmonary microthrombi, and cytokine activation) • Mechanical ventilation • Sepsis • Secondary to left-sided heart failure • RV outflow tract obstruction
**Acute increased volume overload (preload)** • Severe tricuspid regurgitation • Severe pulmonary regurgitation • Intra-cardiac shunt (ASD, VSD)
**Impaired inotropy** • RV myocardial infarct or ischemia • Arrhythmia • Cardiomyopathy, myocarditis • Pericardial disease • Trauma (LVAD or Pacemaker insertion) • After cardiothoracic surgery

## LIST OF ABBREVIATIONS

HFrEF: Heart Failure with Reduced Ejection Fraction
HFpEF: Heart Failure with preserved Ejection Fraction
LVF: Left Ventricular Failure
RVF: Right Ventricular Failure
BVF: Biventricular Failure
NYHA: New York Heart Association
ACC/AHA: American College of Cardiology/American Heart Association
ARF: Acute Heart Failure
ARHF: Acute Right Heart Failure
RVD: Right Ventricular Dysfunction
RV: Right Ventricle
LV: Left Ventricular
PAP: Pulmonary Artery Pressure
MI: Myocardial Infarction
ARDS: Acute Respiratory Distress Syndrome
LVAD: Left Ventricular Assisted Device
BNP: Brain Natriuretic Peptide
NT-proBNP: N-Terminal proBNP
NPV: Negative Predictive Value
CRP: C-reactive Protein
CXR: Chest Radiography
ECG: Electrocardiography
PE: Pulmonary Embolism
MRI: Cardiac Magnetic Resonance Imaging
CT: Computed Tomography
PVR: Pulmonary Vascular Resistance
TAPSE: Tricuspid Annular Plane Systolic Excursion
PASP: Pulmonary Artery Systolic Pressure
MPI: Myocardial Performance Index
FAC: Fractional Area changes
EDD: End-Diastolic Diameter
A4C: Apical four-Chamber
TTE: Transthoracic Echocardiography
TEE: Transesophageal Echocardiography
PLAX: Parasternal Long-Axis
PAP: Pulmonary Artery Pressure
IVC: Inferior Vena Cava
IVS: Interventricular Septum
PSAX: Parasternal Short Axis
RVH: Right Ventricular Hypertrophy
PAT: Pulmonary Acceleration Time
COP: Cardiac Output
CVP: Central Venous Pressure
PEEP: Positive End-Expiratory Pressure
sPAP: Systolic Pulmonary Artery Pressure
RVGLS: Right Ventricle Global Longitudinal Strain
VVI: Velocity Vector Imaging
SR: Strain Rate
CO: Cardiac Output
TR: Tricuspid Regurgitation
